# The Influence of Mediterranean and Western Dietary Patterns on Sensory Perception and Taste Sensitivity: A Study Among University Students

**DOI:** 10.3390/foods14162827

**Published:** 2025-08-15

**Authors:** Ghazal Zolfaghari, María José Castro-Alija, María Laguillo Diaz, Luis Carlos Ramón-Carreira, José María Jiménez, Irene Albertos

**Affiliations:** 1 Recognized Research Group: Multidisciplinary Assessment and Intervention in Health Care and Sustainable Lifestyles, University of Valladolid, 47003 Valladolid, Spain; gzolfaghari@uva.es (G.Z.); luiscarlos.ramon@uva.es (L.C.R.-C.); jose.maria.jimenez@uva.es (J.M.J.); irene.albertos@uva.es (I.A.); 2 Department of Nursing, Faculty of Nursing, University of Valladolid, 47003 Valladolid, Spain; marialaguillodiaz@gmail.com

**Keywords:** dietary adherence, sensory perception, Mediterranean diet, Western diet, taste sensitivity, food preferences

## Abstract

This study examines the relationship between dietary patterns, specifically the Mediterranean diet (MD) and the Western diet (WD), and sensory perception among nursing students at the University of Valladolid, Spain. The study aims to understand how these dietary patterns affect taste sensitivity and preferences, contributing to the fields of nutrition and sensory science. **Materials and Methods:** A total of 41 students participated in this study, following ethical guidelines. Food-grade materials such as refined salt, sucrose, monosodium glutamate, water, and breadsticks were used in sensory assessments. The study involved structured sensory evaluations along with dietary habit questionnaires. Sensory tests were conducted to measure taste perception, and statistical analyses were performed using IBM SPSS Statistics 25.0, with descriptive statistics and correlation analysis. **Results:** The findings revealed significant differences in taste perception across diet adherence levels. Specifically, higher adherence to the MD was associated with a higher perceived intensity and enjoyment of saltiness and umami, while a higher adherence to the WD showed a preference for sweetness. Significant correlations were found between diet adherence and taste enjoyment, with stronger positive associations for saltiness and umami under the MD. **Conclusions:** This study emphasized the impact of dietary habits on taste perception. Adherence to the MD enhanced sensitivity to moderate taste intensities, while adherence to the WD resulted in decreased perception at lower concentrations and heightened sensory responses at higher intensities. These results suggest that long-term dietary patterns influence taste receptor adaptation, potentially affecting food choices and overall health.

## 1. Introduction

Dietary habits are deeply embedded in cultural, social, and cognitive frameworks, playing a significant role in shaping individual health outcomes [1]. These patterns are usually developed during an early age and are often challenging to alter in adulthood [2], indicating the necessity of forming correct eating behaviors from childhood [2,3]. Among the various dietary patterns, the Mediterranean diet (MD) has been acknowledged and valued for its health benefits and protection against chronic diseases [4]. Originating chiefly from Mediterranean countries such as Spain, Italy, and Greece, the MD has been associated with a reduced incidence of cardiovascular diseases, metabolic disorders, and other chronic diseases in the population [5]. The MD prioritizes plant-based foods, including vegetables, fruits, legumes, nuts, moderate amounts of fish, and olive oil as the primary source of fat, with limited consumption of red meat and processed meat [6,7]. Alcohol, particularly wine during meals, is consumed in moderation and is a part of the social and cultural components of eating [8]. Finally, in terms of fat content, the MD is rich in monounsaturated and polyunsaturated fats, particularly omega-3 fatty acids, which are known to reduce the risk of chronic diseases [9].

However, recent data indicate a concerning decline in adherence to the MD, particularly among younger populations in Spain, as they increasingly adopt Westernized eating habits [10]. The Western diet (WD), in contrast to the MD, can be described as containing high levels of sodium, sugars, and unhealthy fats, while having a low fiber content and high levels of ultra-processed products. This dietary pattern is typical of a modern, fast-paced world characterized by stress, insufficient time for cooking, and a desire to consume ready-made foods [10,11]. The WD has also been associated with a range of adverse health conditions such as obesity, type 2 diabetes, cardiovascular diseases, and inflammatory diseases. Furthermore, the sensory-dominant aspects of the WD stimulate craving for salt, sugar, and fat and blur the boundaries of taste preferences, leading to a loss of sensitivity to those flavors [12].

Although Spain is conventionally associated with the MD [5], according to “Per capita consumption data in Spanish households, there have been changes observed in current eating trends in Spain since 2019. The consumption of ultra-processed foods such as pastries, chocolates, and ready-to-cook meals is increasing, while the consumption of key MD elements like vegetables, fruits, and olive oil is decreasing [13]. This aligns with the international trend of consuming less nutritious food and more convenience food, raising concerns about the potential public health impact of these changes [11]. The DELICIOUS project reported that less than 20% of youth meet recommendations for MD and plant-based foods [14].

The MD and WD are two completely different dietary models, each directly impacting health. The MD emphasizes nutrient-rich whole foods, while the WD prioritizes convenience, often sacrificing nutritional quality. This difference can also impact sensory perceptions [15]. For instance, strict adherence to the MD may result in decreased taste preferences for sweet and salty foods. Conversely, the hyper-palatable foods in the WD may desensitize taste receptors and lead to overeating and unhealthy food choices [16].

There are new physiological findings that suggest elements of the WD can alter the brain’s reward circuitry, specifically dopaminergic circuits involved in motivation and cravings. These dietary factors act as additives that increase the desire for hyper-palatable foods, enhancing pleasure responses and reducing the body’s ability to regulate appetite. Over time, this can lead to compulsive eating habits and a decreased response to internal signals of fullness [17,18].

Furthermore, repeated consumption of the WD has been linked to the downregulation of taste receptor genes such as the TAS1R family, which can result in a decreased sensitivity to taste and a higher tolerance for sweet and salty flavors. This reduced nerve sensitivity may lead to a change in anticipation of rewards, ultimately promoting more consumption of highly processed foods [19,20].

In contrast, the MD, with its nutrient-dense and minimally processed nature, can help maintain lower thresholds for detecting saltiness and sweetness, encouraging the consumption of naturally satisfying flavors [21].

It is unclear whether these neuro-biological adaptations affect how individuals perceive the intensity of essential tastes like sweet, salty, or umami. Some research suggests that regular exposure to highly processed foods can lead to sensory desensitization, reducing sensitivity to subtle flavors and potentially reinforcing the preference for highly palatable foods.

Despite the increasing number of studies linking dietary habits to metabolic and behavioral well-being, there is limited concrete research on how adherence to the MD or WD diet can impact sensory perception, especially among young adults in critical stages of behavioral and nutritional development. This study aims to address this research gap by exploring the relationship between adherence to these diets and sensory perception (including sensitivity, thresholds, and hedonic responses) among university students at the University of Valladolid, Spain.

The novelty of this study lies in its interdisciplinary approach, combining validated dietary adherence indexes with objective sensory testing protocols, a method not commonly seen in the existing literature. While previous research has focused on individual dietary components or general taste trials, this study utilizes a multidimensional sensory evaluation model within the context of dietary patterns to provide a comprehensive understanding of how habitual diets can influence taste responses.

This research will offer valuable insights into the connection between diet and sensory perception, particularly among nursing students, shedding light on how diet impacts taste sensitivity and preferences. By studying university students in the stage of solidifying long-term eating habits, this work fills an important gap in the academic literature and addresses the public health implications of dietary changes affecting younger individuals.

Furthermore, the study delves into various hedonic responses and sensory preference data, offering practical insight into leveraging sensory and nutritional strategies to maintain traditional diets and navigate dietary transitions.

In conclusion, this research aims to generate knowledge that supports scientific progress and public health interventions. By emphasizing the biochemical and behavioral interactions between diet and sensory perception, it presents a novel approach to developing effective strategies for improving dietary habits in rapidly changing food environments. This work not only reinforces the cultural and health benefits of the MD but also underscores the importance of countering the sensory and behavioral influences of Westernized eating patterns.

## 2. Materials and Methods

### 2.1. Materials

Refined salt (Sal Costa, Barcelona, Spain), sucrose (Azucarera, Valladolid, Spain), and monosodium glutamate (Ajinomoto, Tokyo, Japan) were purchased from Carrefour in Valladolid, Spain. Spring water (Font Vella, Barcelona, Spain) and breadsticks (Hacendado, Valencia, Spain) were also bought from the same retailer. All materials and equipment used were food-grade. Laboratory tools included disposable plastic cups, weighing trays, a stainless steel spoon, borosilicate test tubes, a handheld stirrer, and a precision digital scale (Fisher Scientific, Barcelona, Spain).

### 2.2. Participants

This study was conducted among nursing students at the University of Valladolid. Participation was voluntary, anonymous, and confidential, with findings utilized solely for research, teaching, and educational purposes. The inclusion criteria required participants to be students at the University of Valladolid who provided informed consent. Exclusion criteria encompassed individuals with food allergies, sensory impairments, metabolic conditions such as diabetes mellitus or hypertension, as well as pregnant or breastfeeding individuals. Ethical approval was granted by the designated institutional review board (Faculty of Nursing’s Ethics Committee at the University of Valladolid, approval date 23 February 2024). The research adhered to a legal framework (Organic Law 3/2018) that protects data privacy (anonymity), participant autonomy (voluntariness), and ethical data use for specific academic purposes. Informed consent, in accordance with established ethical principles (Declaration of Helsinki), was obtained through information sheets and consent forms. Participation was contingent upon understanding the study’s objectives.

### 2.3. Data Collection and Variables

The data for this study were collected using two questionnaires and two sensory tests (see Appendix A). The first questionnaire gathered information on students’ profiles, including their adherence to the MD and consumption of processed foods. Student profiles included variables such as age, sex, residence, physical activity level, weight, height, and food preparation habits. MD adherence was assessed using the PREDIMED questionnaire [22], which consists of 14 questions evaluating the consumption of key MD foods such as olive oil, vegetables, fruits, fish, and legumes. The total score ranges from 0 to 14, classifying adherence levels as high, medium, or low [23]. Additionally, processed food consumption was measured using the SQ-HPF questionnaire [24], which also follows a 14-point scoring system, estimating the percentage of highly processed foods consumed relative to total daily intake in grams [25].

The second questionnaire assessed sensory perception based on the results of two sensory tests. These tests examined the perception of salty, sweet, and umami flavors. Test 1 focused on threshold detection, determining the minimum concentration of a solute required for a student to detect and identify a flavor. Participants were presented with eight samples per flavor, each containing different concentrations (see Table A2 in Appendix B). Samples were arranged from lowest to highest concentration, and students were asked to identify the first glass in which they detected a solute, perceived a change, and could recognize the flavor. To minimize sensory fatigue, as a wash-out session, participants were advised to take breaks between tastings, drink water, and consume breadsticks to neutralize residual taste.

Test 2 aimed to evaluate both flavor intensity and hedonic perception. Participants were presented with three samples per flavor at varying concentrations (See Table A2 in Appendix B). Unlike the threshold detection test, samples were presented in a randomized order. Participants rated intensity on a scale from 1 to 10, where 1 indicated low intensity and 10 indicated high intensity. Hedonic perception was measured using a 9-point scale ranging from “extremely unpleasant” (1) to “extremely pleasant” (9). Finally, students were asked to rank the samples in order of increasing intensity and preference. Similar to the previous test, breaks were recommended between batches to cleanse the palate.

The variables assessed in this study were categorized into two primary groups:

Sample Descriptors: This group includes qualitative variables used to characterize the study population, such as sex, body mass index (BMI), residence, physical activity level, and smoking status.

Response Variables: Adherence to the MD, consumption of processed foods (WD), and response variables, such as detection, identification, intensity, hedonism, and preference.

### 2.4. Statistical Analysis

The behaviors were categorized and compared based on two aspects: intensity and enjoyment regarding the Mediterranean and Western dietary patterns. This analysis was conducted using IBM SPSS Statistics 25.0, incorporating descriptive statistics and correlation analysis to examine how each participant engaged with their assigned dietary patterns and evaluated their perception of flavor intensity and sensory enjoyment.

The Chi-square test was employed to assess the association between diet adherence levels (Mediterranean vs. Western) and categorical taste perceptions (saltiness, sweetness, umami) at various concentrations.

The Kruskal–Wallis test was used to assess differences in taste perception and enjoyment across diet adherence levels (Mediterranean vs. Western).

The Bonferroni correction was applied to adjust the significance threshold for multiple pairwise comparisons between the three adherence levels (1 = low, 2 = medium, and 3 = high) to the MD and WD. With a Bonferroni-adjusted alpha value of 0.0167 (derived from dividing the original significance level of 0.05 by the number of comparisons), the Kruskal–Wallis H test identified significant differences in the perceived intensity and enjoyment of saltiness, sweetness, and umami at varying concentrations (low, medium, and high).

Spearman correlation analyses were performed to assess the relationship between adherence to the MD and the perceived intensity and enjoyment of flavors. Effect sizes were calculated to quantify the magnitude of associations and group differences. For Chi-square tests, Cramér’s V was used to indicate the strength of association [26]. For Kruskal–Wallis tests, eta squared (η^2^) was calculated from the H statistic to estimate variance explained by group differences [27]. Following Bonferroni correction, the effect size (r) was calculated for each pair based on the formula r = Z/√N (where Z is the standardized test statistic (from Mann–Whitney or Wilcoxon test), and N is the total number of observations in the comparison (sum of both groups)). The effect sizes were interpreted using established thresholds proposed by Cohen. For r values and Cramér’s V, the thresholds were small (≈0.1), medium (≈0.3), and large (≥0.5). For η^2^, they were interpreted as small (η^2^ ≈ 0.01), medium (η^2^ ≈ 0.06), and large (η^2^ ≥ 0.14). Reporting effect sizes alongside *p*-values enhances interpretation by showing practical significance [28].

## 3. Results

### 3.1. Demographic Characteristics

The study initially included 42 participants in its statistical population. However, after one participant withdrew consent, the final sample was 41 individuals. The gender distribution was predominantly female, with 29 women (70.73%) and 12 men (29.27%).

In terms of age, the majority (61%) of participants were between 18 and 20 years old, followed by 21–29 years old (22%), 30–49 years old (12%), and a small percentage (5%) were over 50 years old.

When it came to living arrangements, most participants lived with their families (63.41%), followed by those in university residences (19.51%) and student apartments (17.07%).

Analysis of the body mass index (BMI) data showed that most participants had a normal BMI. A smaller portion was categorized as overweight (12.20%), while underweight, Class 1 obesity, and Class 3 obesity each made up 2.44% of the sample.

Physical activity levels varied, with 41.46% engaging in daily exercise, 46.34% participating occasionally, and 12.20% reporting no physical activity.

Smoking behavior was minimal, with only 9.76% of participants identifying as smokers.

### 3.2. Statistical Analysis of Perceived Intensity and Enjoyment of Tastes

#### 3.2.1. Diet Adherence and Taste Perception/Enjoyment

The Chi-square analysis revealed significant relationships between adherence levels to the Mediterranean and Western dietary patterns and the perceived intensity and pleasure of saltiness, sweetness, and umami, with varying intensities observed. These relationships were most pronounced for stimuli of high concentration. Specifically, individuals who adhered more to the MD tended to perceive saltiness and sweetness more strongly at high concentrations (saltiness, *p* < 0.001, sweetness, *p* < 0.001, and umami, *p* = 0.001), while enjoyment levels varied based on taste and concentration. Additional details of these associations, including effect sizes, can be found in Table 1.

Overall, an increase in perception associated with better dietary adherence led to heightened enjoyment, particularly in terms of preference range and intensity level.

#### 3.2.2. Differences in Taste Perception and Enjoyment Among Adherence Groups

The Kruskal–Wallis test further clarified that significant differences existed between adherence groups, particularly in hedonic responses (see Table 2). Interestingly, greater dietary adherence was linked to heightened perception of saltiness and sweetness. However, enjoyment patterns revealed a preference for milder flavors among those with higher adherence to the MD. In contrast, the WD group favored more intense, processed flavors, with significant differences observed in saltiness (*p* = 0.017 for high, *p* = 0.002 for low), sweetness (*p* = 0.003 for low, *p* = 0.008 for medium), and umami (*p* = 0.025 for high, *p* = 0.008 for medium).

These findings suggest that dietary patterns not only influence sensory sensitivity but also modulate taste preferences and hedonic evaluations.

#### 3.2.3. Influence of Diet Adherence on Sensory Perception: Variations in Flavor Intensity and Enjoyment

The Bonferroni correction results, with an adjusted alpha value of 0.0167, revealed that the intensity of flavors, such as saltiness, sweetness, and umami, was influenced by adherence to the diet, although the patterns differed across flavor types. The significant differences observed in the perception of taste intensity and enjoyment at different levels of adherence to the WD and MD are presented in Table 3.

##### Mediterranean Diet (MD)

The analysis of saltiness, sweetness, and umami concentrations revealed that individuals with high adherence to the MD perceived greater intensities for these flavors at higher concentrations. Specifically, for saltiness, a significant difference was noted between high and low adherence groups at high concentrations (*p* = 0.001), as well as between high and moderate groups (*p* = 0.004). Similarly, for sweetness, those with high adherence to the MD reported a higher intensity at high and medium concentrations (*p* = 0.009 and *p* = 0.016, respectively), and for umami, high adherence individuals perceived a stronger intensity at both high (*p* = 0.043) and medium concentrations (*p* = 0.066). These findings suggest that individuals adhering more strictly to the MD may exhibit a heightened sensitivity to flavor intensities, particularly when the concentrations of these flavor profiles are more pronounced.

In terms of flavor enjoyment, the analysis showed that individuals with higher adherence to the MD tended to report lower enjoyment of stronger flavors, particularly at higher concentrations of saltiness, sweetness, and umami. For saltiness, those with high adherence to the MD expressed significantly less enjoyment at high (*p* = 0.017) and low (*p* = 0.002) concentrations compared to their counterparts with low adherence. Similarly, for sweetness, individuals with high adherence reported significantly lower enjoyment at high (*p* = 0.055) and medium (*p* = 0.008) concentrations. For umami, high adherence individuals showed reduced enjoyment at high (*p* = 0.025) and medium (*p* = 0.008) concentrations. These results suggest that the MD, which emphasizes whole, minimally processed foods, may shape an individual’s preference for milder flavors, potentially due to reduced exposure to highly salted, sugary, or artificially flavored foods. This trend reflects a shift in dietary preferences that may be associated with a cultural and physiological adaptation to milder, more natural flavor profiles typical of the Mediterranean dietary pattern.

##### Western Diet (WD)

For saltiness, significant differences in perceived intensity were observed between the low and moderate adherence groups for both high and medium concentrations (*p* = 0.001 and *p* = 0.003, respectively), but there were no further significant changes between the moderate and high adherence groups. This suggests that adherence to the WD decreases sensitivity to the intensity of saltiness, particularly between the low and moderate groups, but not at higher levels of adherence. Similarly, for sweetness, a decrease in intensity perception was noted as adherence increased, with significant reductions between low and moderate adherence groups (*p* = 0.008 and *p* = 0.003) for high and medium sweetness concentrations. However, the intensity of low sweetness did not vary significantly across adherence levels. Umami, on the other hand, did not exhibit significant changes in intensity across different adherence levels, indicating that individuals adhering to the WD did not experience differences in the perceived strength of umami flavors.

Enjoyment of flavors, particularly saltiness, sweetness, and umami, also exhibited distinct patterns depending on the level of adherence to the WD. For saltiness, enjoyment was significantly higher for low adherence individuals at low concentrations (*p* = 0.000), but there were no significant differences in enjoyment for high and medium saltiness between the groups, suggesting that enjoyment of more intense saltiness does not change with higher adherence. For sweetness, enjoyment was greater for medium sweetness concentrations in the moderate adherence group compared to low adherence (*p* = 0.002), indicating that moderate adherence is associated with a preference for sweeter flavors. No significant differences were found in enjoyment at high or low sweetness concentrations, suggesting stable enjoyment across adherence levels for these intensities. Umami flavors showed a more pronounced pattern, with individuals in the high adherence group enjoying high umami significantly more than those in the low adherence group (*p* = 0.005), and moderate adherence resulted in greater enjoyment of medium umami compared to low adherence (*p* = 0.003). This suggests that higher adherence to the WD may enhance the enjoyment of more intense umami flavors. Overall, the enjoyment of flavors such as saltiness and sweetness appeared to be influenced by moderate adherence to the WD, while umami enjoyment was notably higher for those with greater adherence.

#### 3.2.4. Correlation Between Taste Perception/Enjoyment and Diet Adherence

##### Mediterranean Diet (MD)

Table 4 demonstrates that significant positive correlations were found between adherence to the MD and perceived intensity of high saltiness (ρ = 0.579), sweetness (ρ = 0.485), and umami (ρ = 0.378). Enjoyment was positively correlated with lower saltiness (ρ = 0.548) and umami (ρ = 0.563), and negatively correlated with low sweetness (ρ = −0.363), suggesting a preference for a more refined flavor profile. In conclusion, individuals who closely adhere to the MD show a preference for both high-intensity and low-intensity flavors, particularly favoring low saltiness and umami, while also preferring lower sweetness.

##### Western Diet (WD)

The WD exhibited a different pattern. Higher adherence was linked to a heightened perception of intense saltiness, sweetness, and umami. However, enjoyment leaned towards more intense flavors.

Individuals who closely follow the WD typically have a heightened taste perception, especially for high levels of saltiness, sweetness, and umami. This is supported by moderate positive correlations of 0.558, 0.504, and 0.424, respectively. In terms of flavor enjoyment, those on the WD have a strong preference for higher-intensity sweetness, with very strong positive correlations for both high sweetness (0.854) and medium sweetness (0.994). Conversely, there is a moderate negative correlation (−0.427) for enjoyment of low saltiness, indicating less enjoyment of low-intensity flavors. In conclusion, individuals on the WD favor intense flavors, particularly high sweetness, while showing less enjoyment for low-intensity flavors like low saltiness.

#### 3.2.5. Correlation Between BMI, Age, and Taste Perception Based on Dietary Pattern and Smoking Status

The study examined the correlations between BMI, age, and taste perception (saltiness, sweetness, and umami at high, medium, and low intensities) in relation to smoking status (non-smokers and smokers) and dietary patterns (MD and WD). Among non-smokers, BMI had weak or negligible correlations with taste perception across all intensities, with coefficients ranging from approximately −0.13 to 0.16. These results suggest that there is no significant link between BMI and taste sensitivity in non-smokers. However, age showed a consistently moderate negative correlation with low-intensity umami perception (r ≈ −0.45), indicating that older age may be associated with reduced sensitivity to subtle umami flavors, regardless of dietary patterns. Correlations between age and saltiness or sweetness perception were generally weak and inconsistent. Conversely, among smokers, the correlation patterns were highly variable and inconsistent. Some strong correlations were observed (such as between BMI and high-intensity sweetness in the MD group (r = −0.95), but due to the small sample size, these results should be interpreted cautiously. Overall, the findings suggest that while BMI does not significantly impact taste perception in non-smokers, age may play a role in reducing sensitivity to low-intensity umami tastes (Table 5).

### 3.3. Descriptive Reporting of Median Data on Intensity and Enjoyment of Tastes

To provide a comprehensive representation of the patterns in perceived flavor intensity and enjoyment, the following graphs illustrate the median values of participant responses across different concentration levels and adherence categories for the MD and WD.

**Figure 1:**
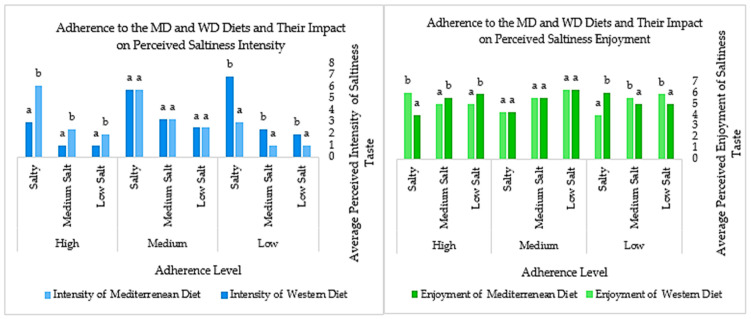
Trends in saltiness taste intensity and enjoyment across MD and WD adherence. (Values (mean ± standard deviation) identified with different letters have significant differences between them (*p* < 0.05). Values (mean ± standard deviation) identified with the same letter do not have significant differences between them (*p* > 0.05)).

Figure 1 reveals that the closer one adheres to the MD, the more pronounced the trend of perceiving each flavor’s intensity, especially at higher salt concentrations. Enjoyment perception, on the other hand, shows an inverse relationship with salt concentration, with individuals with higher adherence rates experiencing reduced enjoyment as salt levels increase. Individuals following the WD tend to have lower enjoyment at lower salt levels with low adherence, but peak enjoyment is reached at higher salt levels with high adherence. In contrast, those who follow the MD experience stronger intensity and greater enjoyment at lower salt amounts compared to WD followers, who experience increased intensity and enjoyment at higher salt levels.

**Figure 2:**
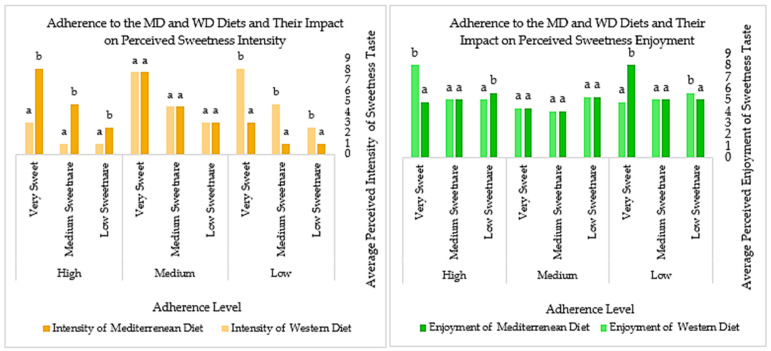
Trends in sweetness taste intensity and enjoyment across MD and WD adherence. (Values (mean ± standard deviation) identified with different letters have significant differences between them (*p* < 0.05). Values (mean ± standard deviation) identified with the same letter do not have significant differences between them (*p* > 0.05)).

The trend shown in Figure 2 for sweetness is similar to the trend for saltiness. As adherence to the MD increases, the perception of sweetness intensity becomes more pronounced, especially at higher concentrations, while enjoyment tends to decline with increasing sweetness. On the other hand, in the WD, enjoyment is lower at mild sweetness levels for those with low adherence but peaks at higher sweetness concentrations for those with high adherence. This pattern is consistent with the trend observed for saltiness, where MD followers perceive stronger intensity but prefer lower concentrations, while WD followers experience greater enjoyment at higher intensities.

**Figure 3:**
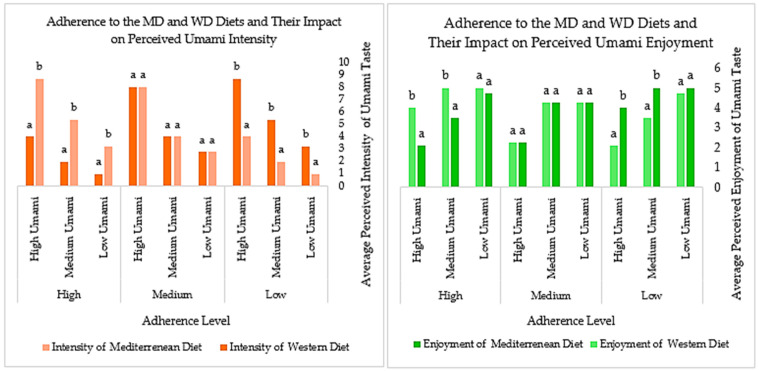
Trends in umami taste intensity and enjoyment across MD and WD adherence. (Values (mean ± standard deviation) identified with different letters have significant differences between them (*p* < 0.05). Values (mean ± standard deviation) identified with the same letter do not have significant differences between them (*p* > 0.05)).

Based on Figure 3, a similar trend can be observed in the perception of umami. The umami taste is primarily driven by monosodium glutamate, which is commonly found in many processed foods. Individuals with higher adherence to a WD may be more accustomed to this flavor due to its frequent presence in their typical food choices, potentially influencing both their intensity perception and enjoyment of umami.

## 4. Discussion

This study investigated the relationship between dietary patterns (MD and WD) and sensory perceptions among nursing students at the University of Valladolid, Spain. The findings reveal differences in sensitivity and enjoyment of saltiness, sweetness, and umami, influenced by adherence levels to these diets. Adherence to the WD was associated with reduced sensitivity to these tastes, particularly at lower concentrations, supporting the idea that habitual consumption of hyper-palatable foods high in salt, sugar, and fat can lead to diminished taste receptor sensitivity [29]. These findings contribute to understanding the interaction between dietary patterns, sensory adaptation, and potential public health implications. For example, high WD adherence also correlated with a preference for intensely flavored foods, suggesting a hedonic shift that may drive overconsumption and elevate risks of obesity and metabolic disorders [30]. In contrast, MD adherence was linked to heightened sensitivity to saltiness, sweetness, and umami, especially at higher concentrations, likely due to the diet’s nutrient-dense and minimally processed characteristics that help preserve taste receptor functionality. The MD also promoted a preference for moderate flavor intensities, which may aid satiety and prevent overconsumption, reinforcing its association with improved weight management and reduced chronic disease risk.

These findings are consistent with studies that found young adults in the Mediterranean region, who prefer Mediterranean foods, are more likely to adhere to the MD, make healthier food choices, and consume fewer sweet, fatty, and processed foods [31,32]. Additionally, an intervention study showed that a hypocaloric MD can increase sensitivity to salt taste in obese patients, highlighting the impact of diet on taste preferences [33,34]. Other studies have shown that MD adherence is associated with higher consumption of fruits, vegetables, whole grains, and nuts, and lower consumption of refined grains, red meats, and processed foods, leading to positive health outcomes [35].

Overall, these findings suggest that traditionally and minimally processed dietary patterns help preserve taste preferences and reduce the likelihood of excessive eating and metabolic disease associated with the WD’s hyper-palatable nature.

Physiologically, these patterns may stem from both dietary and host factors. Chronic WD exposure may downregulate taste receptor expression (such as the TAS1R family) [19], flattening neural gustatory responses and shifting reward thresholds for taste stimuli [20]. Conversely, MD’s nutrient density and diversity help sustain taste papillae function, supporting lower salt and sweet thresholds and favoring satiety-inducing flavor profiles [21]. Our data are consistent with short-term dietary interventions, and the results of these studies based on that reduction in sodium intake via MD protocols indicate a sensitization of salt taste receptors, modulating food preferences and taste detection thresholds. To better understand the biochemical pathway in this area, extensive biochemical and physiological studies are also needed.

Demographic and lifestyle factors, such as living arrangements and BMI [36], appear to influence dietary adherence [37] and sensory perception. Individuals living with family may have greater exposure to traditional dietary patterns like the MD compared to those living independently, suggesting that social and environmental factors play a critical role in shaping dietary behaviors [38,39]. Due to limitations in sample size and an uneven distribution of participants across lifestyle variables, this study was unable to robustly assess the influence of factors such as cohabitation status (living with family versus alone) [40] and physical activity on taste perception [41]. These factors have been suggested to modulate sensory experiences and dietary behavior through mechanisms related to psychological well-being, social engagement, and metabolic health [40,41]. Therefore, we could not determine if our conclusions were consistent or inconsistent with previous studies, particularly that of Gauthier et al. [41], on the impact of an active lifestyle on the preservation of specific taste perceptions. Future research with larger, stratified cohorts is needed to explore these associations more comprehensively and to clarify the potential interactive effects between psychosocial and behavioral determinants on taste perception.

Furthermore, BMI impacts sensory enjoyment, with overweight or obese individuals showing altered sensory responses, potentially due to hormonal and neurological changes associated with obesity [42,43].

While Vignin et al. and Hwang et al. indicate a general decrease in taste sensitivity with increased BMI, suggesting that higher BMI may impair taste perception and potentially influence eating behavior and weight management strategies [44,45]. Results from our study in non-smokers showed weak and inconsistent associations between BMI and taste perception across all taste modalities and intensity levels, which were not significant.

Conversely, age consistently showed a moderate negative correlation with low-intensity umami perception, indicating an age-related decline in the ability to detect subtle umami stimuli. Correlations between age and other taste qualities, such as saltiness and sweetness, were minimal and did not show a discernible trend.

This finding supports existing evidence from the Vignin et al. study of sensory decline with aging, particularly in complex taste modalities like umami [44].

Among smokers, the correlation patterns were markedly inconsistent and, in some cases, extreme—such as the strong inverse correlation between BMI and high-intensity sweetness perception in the Mediterranean diet subgroup, aligning with previous literature suggesting that the correlation between smoking and taste perception is inconsistent, with some extreme cases observed [46]. Smoking is known to impair taste receptor function, yet the present data are insufficient to draw definitive conclusions. The limited number of smokers also prevents significant conclusions regarding the influence of smoking on taste perception. Overall, while BMI appears to have minimal influence on taste perception in non-smokers, aging may selectively affect umami sensitivity, and the low prevalence of smoking intentions among participants suggests a health-conscious sample, which may not reflect the general population. Further research with larger, more stratified cohorts is essential to elucidate the complex interactions between age, body composition, smoking behavior, and sensory function.

The restriction to nursing students, who are predominantly female and from a Mediterranean setting, potentially limits the generalizability of our results, as these individuals are likely more health-literate than their peers.

Hormonal fluctuations, particularly those related to the menstrual cycle, may influence taste perception, especially sensitivity and preference for sweetness and saltiness [47,48]. This is especially important given the gender imbalance in our sample (70% female), which might have introduced confounding effects independent of dietary pattern.

Although the choice of monosodium glutamate (MSG) to enhance umami perception taste is a promising strategy [49], it is not the optimal way to evaluate the quality of umami in a natural MD environment [50,51]. Umami is typically found in a food matrix (such as tomatoes or mature cheese) [51] in this context, which could potentially skew the results. Some consumers view MSG as an ‘unhealthy’ and ‘artificial’ food additive [52]. MSG has been associated with obesity, metabolic disorders, Chinese Restaurant Syndrome, neurotoxic effects, and negative impacts on reproductive organs [53]. A study by Wang and Adhikari [54] revealed that over 60% of consumers in the United States try to avoid or reduce their consumption of MSG-containing foods, despite the fact that the FAO and WHO have deemed MSG safe for consumption. Future studies should incorporate natural taste stimuli that are environmentally related and have been established as safe and healthy by both the general public and the scientific community.

From a public health perspective, promoting the MD, especially among young adults transitioning to independent living, is crucial. Sensory education and strategies to reduce the consumption of ultra-processed foods could mitigate sensory desensitization and foster healthier eating habits [16]. Additionally, incorporating sensory science into dietary guidelines and public health initiatives could enhance their effectiveness by addressing the sensory and hedonic factors influencing food choices [55,56].

However, these results provide some directions for further investigation and practice in public health based on the main findings: causality should be confirmed, and generalization should be expanded through larger, longitudinal, multi-center studies. Studies should include stratification by gender, field of study, smoking habits, level of physical activity, lifestyle, and place of residence. These factors are highly recommended for further research. The validity of sensory assessments can be enhanced by measuring other modalities (bitter, sour, fat) as well as using real food materials instead of tastant solutions. To address demographic confounders, it is important to include covariates on hormonal status and nutrition knowledge. Sensory teaching and precision nutrition interventions that focus on taste adaptation and food preferences can promote healthier eating habits and long-lasting weight management. Longitudinal studies are necessary to determine if taste sensitivities modulated through dietary intervention are sustained and if this modulation leads to improved adherence and reduced disease pathologies.

In summary, our report contributes to the growing body of data suggesting that the MD maintains taste receptor physiology and encourages healthy food choices, potentially playing a role in maintaining healthy body weights and preventing chronic illnesses. Conversely, adherence to a WD may lead to sensory numbing, overconsumption of food, and metabolic risks in the future. Addressing behavioral and sensory issues in dietary regimens and community health initiatives is crucial for evidence-based planning.

## 5. Conclusions

The results suggest that high adherence to the Mediterranean diet is associated with increased sensitivity to tastes at high concentrations, indicating a preference for balanced and less intense flavors. In contrast, individuals with greater adherence to the Western diet showed reduced perception at lower concentrations and stronger hedonic responses at higher concentrations, possibly indicating a preference for more intense, hyper-palatable flavors linked to processed foods.

Dietary habits not only affect taste preferences but also impact overall sensory enjoyment. Followers of the MD experienced peak enjoyment at lower concentrations, which decreased with increasing intensity, showing a preference for natural and moderate flavors. On the other hand, followers of the WD enjoyed stronger flavors, especially at higher concentrations, suggesting the influence of a diet focused on convenience and intense flavors.

These findings underscore the broader implications of dietary habits on health. The MD promotes a balanced approach to flavor and nutrition, while the WD may lead to desensitization and unhealthy eating behaviors. This study highlights the significant role of dietary habits in shaping health and the sensory experience of food.

The results stress the importance of promoting dietary patterns like the MD as a strategy to maintain or restore healthy taste perception. Public health interventions should include sensory education to help individuals, especially youth, adjust their taste preferences towards less processed and more nutrient-dense foods. Policymakers and educators could implement initiatives that limit youth exposure to ultra-processed foods and support food environments that prioritize natural flavor experiences. These efforts may prevent long-term desensitization to basic tastes and reduce the appeal of nutritionally poor but highly stimulating foods.

However, these findings should be interpreted cautiously due to several limitations, including the small sample size, limited age range, and relatively homogeneous population, which may limit the generalizability of the results.

Future research should aim to replicate these findings in larger, more diverse populations and include objective physiological or biochemical measures, such as taste receptor expression or salivary markers. Longitudinal and intervention-based studies are also necessary to determine whether dietary modifications can reverse taste desensitization and restore healthier taste preferences over time. Understanding these mechanisms could inform public health strategies aimed at promoting sustainable dietary changes and improving both nutritional status and food enjoyment.

## Figures and Tables

**Table 1:** Diet adherence and taste perception.

Perception	Taste Type	Concentration	Chi-Square (χ^2^)	df	*p*-Value	Effect Size
Intensity	Saltiness	High	55.07	2	<0.001 ***	0.820
		Medium	25.07	8	0.002 **	0.553
		Low	14.49	6	0.025 *	0.420
	Sweetness	High	29.10	5	<0.001 ***	0.596
		Medium	26.78	6	<0.001 ***	0.571
		Low	24.42	5	<0.001 ***	0.546
	Umami	High	19.73	5	0.001 ***	0.491
		Medium	19.85	4	0.001 ***	0.492
		Low	18.51	7	0.010 **	0.475
Enjoyment	Saltiness	High	34.37	9	<0.001 ***	0.647
		Medium	30.22	7	<0.001 ***	0.607
		Low	25.71	4	<0.001 ***	0.560
	Sweetness	High	24.63	8	0.002 **	0.548
		Medium	18.90	7	0.008 **	0.480
		Low	31.44	5	<0.001 ***	0.619
	Umami	High	33.39	9	<0.001 ***	0.638
		Medium	11.10	7	0.134	0.368
		Low	28.59	8	<0.001 ***	0.590

*p*-Values indicate significance levels: *** *p* ≤ 0.001; ** *p* < 0.01; * *p* < 0.05. Effect sizes > 0.7 should be interpreted cautiously due to small sample size.

**Table 2:** Kruskal–Wallis test results for diet adherence and intensity/enjoyment of taste at different concentrations.

Taste Type	Concentration	Intensity Perception (H, *p*-Value, η^2^)	Enjoyment (H, *p*-Value, η^2^)
Saltiness	High	13.408, 0.001 ***, η^2^ = 0.300	8.156, 0.017 *, η^2^ = 0.162
	Medium	11.261, 0.004 **, η^2^ = 0.244	4.564, 0.102, η^2^ = 0.067
	Low	5.067, 0.079, η^2^ = 0.081	12.005, 0.002 **, η^2^ = 0.263
Sweetness	High	9.466, 0.009 **, η^2^ = 0.196	5.800, 0.055, η^2^ = 0.100
	Medium	8.299, 0.016 *, η^2^ = 0.166	9.695, 0.008 **, η^2^ = 0.203
	Low	3.324, 0.190, η^2^ = 0.035	11.499, 0.003 **, η^2^ = 0.250
Umami	High	6.271, 0.043 *, η^2^ = 0.112	7.378, 0.025 *, η^2^ = 0.142
	Medium	5.444, 0.066, η^2^ = 0.091	9.550, 0.008 **, η^2^ = 0.199
	Low	5.783, 0.055, η^2^ = 0.100	8.035, 0.018 *, η^2^ = 0.159

H: Kruskal–Wallis test statistic. *p*-Values indicate significance levels: *** *p* ≤ 0.001; ** *p* < 0.01; * *p* < 0.05. η^2^ values represent effect sizes for Kruskal–Wallis tests (H/n-). Values ≥ 0.14 indicate large effects; interpret with caution due to small sample size.

**Table 3:** Significant pairwise differences in flavor intensity and enjoyment by diet adherence levels.

		Western Diet			Mediterranean Diet		
Taste Type	Measure (Concentration)	Significance Comparison (Adj. *p*)	[Z-Value]	Effect Size [r]	Significance Comparison (Adj. *p*)	[Z-Value]	Effect Size [r]
Saltiness	High Intensity	Low vs. Medium (0.001 ***)	3.296	0.52	Low vs. High (0.001 ***)	3.296	0.52
	Medium Intensity	Low vs. Medium (0.003 **)	2.953	0.46	Medium vs. High (0.003 **)	2.953	0.46
	Enjoymentow Intensity	Low vs. Medium (<0.001 ***)	3.085	0.48	Low vs. High (0.002 **)	3.085	0.48
	EnjoymentHigh Intensity	–	–	–	Low vs. High (0.017 *)	2.396	0.37
Sweetness	High Intensity	Low vs. Medium (0.008 **)	2.665	0.42	Medium vs. High (0.008 **)	2.340	0.37
	Medium Intensity	Low vs. Medium (0.019 *)	2.340	0.37	–	–	–
	EnjoymentMedium Intensity	Low vs. Medium (0.002 **)	2.112	0.33	–	–	–
	EnjoymentLow Intensity	–	–	–	Medium vs. High (<0.001 ***)	2.996	0.47
	EnjoymentHigh Intensity	–	–	–	Low vs. High (0.016 *)	2.198	0.34
Umami	EnjoymentHigh Intensity	Low vs. High (0.005 **)	1.668	0.26	Medium vs. High (0.003 **)	2.762	0.43
	EnjoymentMedium Intensity	Low vs. Medium (0.003 **)	1.451	0.23	Low vs. High (0.047 *)	2.762	0.43
		Low vs. High (0.028 *)	1.574	0.25	–	–	–
	EnjoymentLow Intensity	–	–	–	Medium vs. High (0.021 *)	2.307	0.36

*p*-Values indicate significance levels: *** *p* ≤ 0.001; ** p < 0.01; * *p* < 0.05.

**Table 4:** Correlation between diet adherence and flavor perception and enjoyment.

Diet Type	Taste Type	Concentration	Intensity Correlation	Enjoyment Correlation
Mediterranean	Saltiness	High	0.579	–
		Low	–	0.548
	Sweetness	High	0.485	–
		Medium	–	0.484
		Low	–	−0.363
	Umami	High	0.378	–
		Low	–	0.563
Western	Saltiness	High	0.558	–
		Low	–	−0.427
	Sweetness	High	0.504	–
		High	–	0.854
		Medium	–	0.994
		Low	–	0.541
	Umami	High	0.424	–
		Low	–	0.454

**Table 5:** Correlation between BMI, age, and taste perception according to dietary pattern and smoking status.

Smoking Status	Diet Type	Characteristic	Saltiness (HighMedLow)	Sweetness (HighMedLow)	Umami (HighMedLow)
Non-Smokers	Mediterranean	BMI	0.16(−0.02)0.05	−0.10–(−0.09)–(−0.02)	0.19–0.16–0.11
		Age	0.00–0.16–(−0.09)	0.09–(−0.14)–(−0.04)	−0.26–(−0.18)–(−0.45)
	Western	BMI	−0.13–(−0.02)–0.05	−0.10–(−0.09)–(−0.02)	0.19–0.16–0.11
		Age	−0.03–0.16–(−0.09)	0.09–(−0.14)–(−0.04)	−0.26–(−0.18)–(−0.45)
Smokers	Mediterranean	BMI	−0.40–(−0.11)–0.11	−0.95–(−0.32)–0.63	−0.95–(−0.95)–(−0.78)
		Age	0.26–0.27–0.82	0.00–0.82–0.00	−0.27–0.00–0.33
	Western	BMI	−0.63–(−0.11)–0.11	−0.95–(−0.32)–0.63	−0.95–(−0.95)–(−0.78)
		Age	0.00–0.27–0.82	0.00–0.82–0.00	−0.27–0.00–0.33

## Data Availability

The original contributions presented in the study are included in the article; further inquiries can be directed to the corresponding author.

## Appendix A. Dissolution Table Test 1

**Table A1:** Dissolutions Test 1: Flavor, compound, and concentration.

Flavor	Salty		Sweet		Umami	
Code	901		290		723	
Batch	1st		2nd		3rd	
Compound	NaCl		Saccharose		Glutamate	
	Sample No.	[g/L]	Sample No.	[g/L]	Sample No.	[g/L]
	980	0.16	330	0.34	600	0.09
	376	0.24	456	0.55	209	0.12
	697	0.34	993	0.94	951	0.17
	125	0.48	105	1.56	118	0.24
	499	0.69	188	2.59	189	0.34
	785	0.98	215	4.32	477	0.49
	348	1.40	233	7.20	734	0.70
	523	2.00	843	12.00	405	1.00

## Appendix B. Dissolution Table Test 2

**Table A2:** Dissolutions. Test 2: Flavor, compound, and concentration.

Flavor	Salty		Sweet		Umami	
Code	901		290		723	
Batch	1st		2nd		3rd	
Compound	NaCl		Saccharose		Glutamate	
	Sample No.	[g/L]	Sample No.	[g/L]	Sample No.	[g/L]
	568	0.16	890	0.94	345	0.12
	620	0.34	263	4.32	123	0.34
	190	0.98	548	12	433	1
